# The Role of Common Solvents against Pseudomonas aeruginosa-Induced Pathogenicity in a Murine Burn Site Infection Model

**DOI:** 10.1128/spectrum.00233-21

**Published:** 2021-08-04

**Authors:** Vijay K. Singh, Marianna Almpani, Laurence G. Rahme

**Affiliations:** a Department of Surgery, Harvard Medical School and Massachusetts General Hospital, Boston, Massachusetts, USA; b Shriners Hospitals for Children Boston, Boston, Massachusetts, USA; c Department of Microbiology, Harvard Medical School, Boston, Massachusetts, USA; Emory University School of Medicine

**Keywords:** DMSO, PEG, PEG400, excipient, *Pseudomonas aeruginosa*, intestinal permeability, intestinal inflammation, virulence, antivirulence

## Abstract

Dimethyl sulfoxide (DMSO) and polyethylene glycols (PEGs) are frequently used as potent excipients in pharmaceutical formulations. However, these agents also have an interesting antimicrobial and anti-inflammatory profile that could interfere with the efficacy testing of anti-infective compounds when the latter are solubilized in DMSO or PEGs. Here, we demonstrate the antimicrobial and anti-inflammatory effects of DMSO-PEG400 in a murine Pseudomonas aeruginosa infection model, aiming to draw attention to the appropriate selection of solvents for difficult-to-solubilize anti-infectives.

**IMPORTANCE** Our study demonstrates the antimicrobial and anti-inflammatory effects of the combination of DMSO and PEG400 against Pseudomonas aeruginosa *in vitro* and *in vivo* in a murine infection model of heightened intestinal permeability. The aim of this study is to draw attention to the appropriate selection of solvents for difficult-to-solubilize anti-infective compounds, to avoid interference with the assay or system tested. This is an extremely important consideration, since potential antimicrobial and anti-inflammatory effects of the solvent vehicle are detrimental to research studies on the efficacy of new anti-infective agents, given that the vehicle effect can mask the effect of the tested compounds. Our results can therefore be of great value to the scientific community, as they can guide researchers in the future to avoid this significant pitfall that can cost substantial amounts of money and valuable time during investigations of the effects of novel, difficult-to-solubilize antimicrobial compounds.

## INTRODUCTION

Dimethyl sulfoxide (DMSO) and polyethylene glycols (PEGs) have long been used as versatile excipients in the formulation of pharmaceutical agents (1) (https://www.accessdata.fda.gov/scripts/cder/iig/index.cfm?event=BasicSearch.page, search for inactive ingredient name: dimethyl sulfoxide; polyethylene glycol). They have frequently been used to improve the solubility of compounds that are difficult to solubilize in water or aqueous solvents (2–4). Even though seemingly alluring for solubility purposes, these agents individually have been shown to possess antimicrobial (4–10) (https://www.dmso.org/articles/information/herschler.htm) and anti-inflammatory properties (6, 11, 12). More specifically, DMSO has been suggested to exert its antimicrobial effects as a result of bacterial membrane penetration and perturbation (13, 14), while PEGs are thought to lead to bacterial cell clumps and microbial morphological alterations that eventually lead to bacterial killing (7). This profile might make them less favorable when used as excipients of new compounds that need to be tested for their antimicrobial efficacy. This problem becomes even more prevalent if the tested compound is expected to affect bacterial virulence without exhibiting bactericidal or bacteriostatic properties. Solubilizing such compounds in DMSO or PEGs could significantly compromise the anti-virulent compounds’ efficacy testing.

Our group has developed a new family of antimicrobial compounds against Pseudomonas aeruginosa (PA) that target bacterial virulence (15–19). To solubilize these novel compounds, we used a combination of DMSO and PEG400 to assess their effects in a clinically relevant murine PA infection model. This combination of excipients was chosen over a number of other options because it provided the best solubility for our compounds. The DMSO-PEG400 combination in the vehicle control group revealed an auspicious role in host intestinal permeability, intestinal inflammation, and systemic bacterial dissemination. Here, we aimed to demonstrate the DMSO-PEG400 antimicrobial and anti-inflammatory effects *in vivo* and draw attention to the appropriate selection of solvents for difficult-to-solubilize anti-infective compounds to avoid interference with the assay or system tested.

## METHODS AND RESULTS

### Effect of DMSO-PEG400 on bacterial growth *in vitro*.

**FIG 1:**
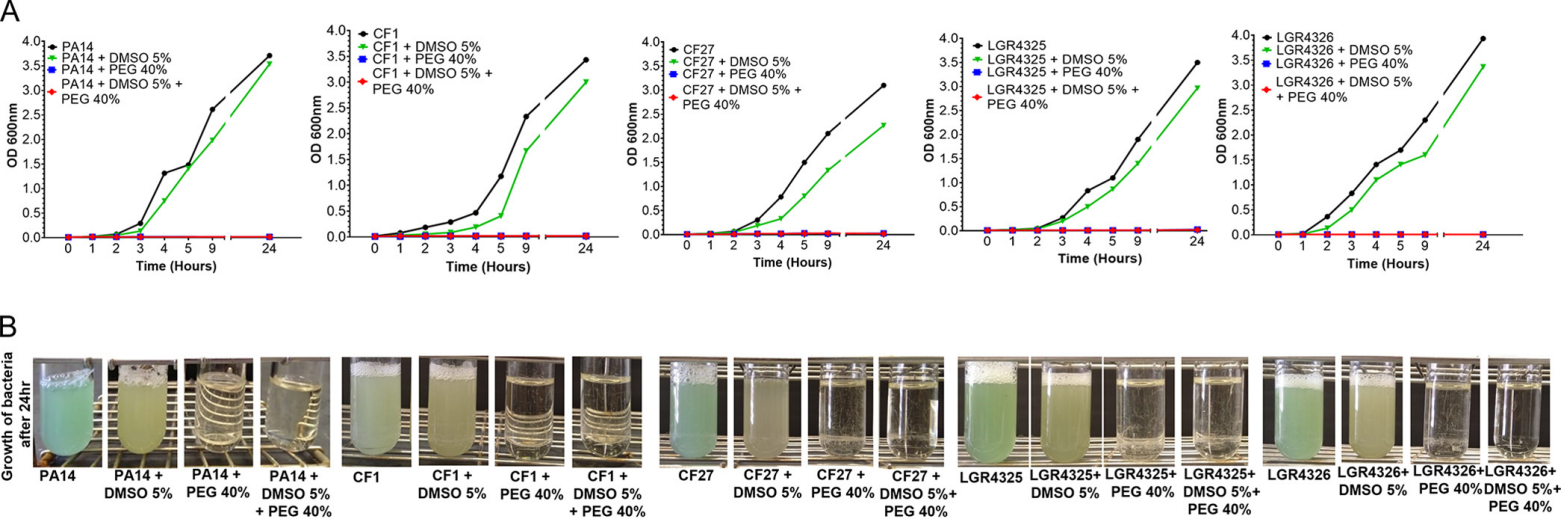
*In vitro* effect of the DMSO-PEG400 combination on the growth of PA strains. (A) Bacterial growth curves in the presence of 5% DMSO, 40% PEG400, or 5% DMSO–40% PEG400. PEG400 and DMSO-PEG400 eliminated bacterial growth *in vitro*. DMSO also reduced bacterial growth compared to the no-treatment group, albeit without the dramatic effect that PEG400 and the solvent combination have. (B) Visual inhibition of bacterial growth in all three conditions.

To understand the DMSO-PEG400 effect on PA growth, we interrogated how it affects bacterial growth *in vitro*. For this assessment, we used five different PA clinical isolates: PA14, a UCBPP-PA14 rifampin-resistant PA human clinical isolate (Rahme laboratory) (20); CF1, a human clinical isolate from a cystic fibrosis patient (obtained from Bonnie Ramsey, Seattle Children’s Hospital, via Stephen Lory); CF27, a human clinical isolate from a cystic fibrosis patient (obtained from Bonnie Ramsey, Seattle Children’s Hospital, via Stephen Lory); LGR4325, a human clinical isolate from a burn patient at the Shriners Hospital for Children (Boston, MA); and LGR4326, a human clinical isolate from a burn patient at the Shriners Hospital for Children (Boston, MA). A single colony of each PA strain grown on Luria-Bertani (LB) agar was inoculated in 5 ml LB broth and was incubated overnight (incubator-shaker; 37°C; 200 rpm). DMSO (5%) alone, PEG (40%) alone, and DMSO (5%)-PEG (40%) were prepared in 5 ml LB broth. For each condition, cultures were left to grow overnight (incubator-shaker; 37°C; 200 rpm) in triplicate. These cultures were diluted to an optical density at 600 nm (OD_600_) of 0.01 and were used as starter cultures, which were incubated (incubator-shaker; 37°C; 200 rpm). Their OD_600_ was measured at different time points, and the growth curve was plotted using GraphPad Prism (GraphPad Software, La Jolla, CA, USA). Figure 1A and B show that addition of 5% DMSO–40% PEG400 in the PA cultures completely halted bacterial growth. PEG400 (40%) alone exerted a similar effect. DMSO (5%) alone also reduced the bacterial growth, but to a lesser extent.

The MIC of DMSO for PA *in vitro* was already available in the literature (8% DMSO) (21). We therefore subsequently sought to determine the MIC of PEG400. A single PA14 colony was inoculated in 5 ml of Mueller-Hinton medium and was incubated overnight (incubator-shaker; 37°C; 200 rpm). The overnight culture was then diluted to an OD_600_ of 0.1 and was used as the starter culture. Different PEG400 concentrations (10%, 15%, 20%, 25%, 30%, 35%, 40%, 45%, and 50%) were added in different tubes with starter culture. The tubes were incubated for 24 h (incubator-shaker; 37°C; 200 rpm), and following incubation, the culture OD_600_ was determined. Based on this assay, the MIC for PEG400 was determined to be the 35% concentration.

### Effects of DMSO-PEG400 treatment *in vivo*.

For the *in vivo* studies, 10-week-old male C57BL/6 mice (The Jackson Laboratory) were maintained in a specific-pathogen-free environment at the Massachusetts General Hospital (MGH), in a 12-h-light–12-h-dark photoperiod, at an ambient temperature of 22 ± 1°C, with food and water access *ad libitum*. Animal protocols were approved by the Institutional Animal Care and Use Committee (IACUC) at MGH (protocol no. 2006N000093) and are in strict accordance with the guidelines of the Committee on Animals of MGH, Harvard Medical School, and the regulations of the Subcommittee on Research Animal Care of MGH and the National Institutes of Health.

Mice were anesthetized (500 μl intraperitoneal injection: ketamine [125 mg/kg] and xylazine [12.5 mg/kg] in normal saline [NS]), and the dorsal fur was removed with a clipper. A 30% total body surface area (TBSA) dorsal burn was induced by immersion in 90°C water for 8 s, using a polystyrene foam template, as described in reference 22, with some modifications. For spinal protection, a dorsal subcutaneous injection of 500 μl NS was administered prior to burn induction. For fluid resuscitation, 500 μl NS was injected intraperitoneally.

Bacteria were grown in LB broth or LB agar plates. One hundred microliters of 10 mM MgSO_4_ containing approximately 3 × 10^5^ CFU of PA14 was intradermally injected at the burn eschar in the burn-plus-infection (BI) group. Mice in the no-infection group received 100 μl of 10 mM MgSO_4_. For the preparation of every 1,000 μl of the 5% DMSO–40% PEG400 combination, 550 μl of distilled water, 50 μl of DMSO (Sigma-Aldrich; lot number SHBF7360V; catalog number D818-50mL; nonexpiring) and 400 μl of PEG400 (EMD Millipore Corporation; lot number S7263585626; catalog number 074851000; nonexpiring) were mixed in a sterile tube. One hundred microliter of 5% DMSO–40% PEG400 was administered subcutaneously at 1, 3, 5, 7, 11, 15, and 19 h post-BI. (Given the fact that the 5% DMSO–40% PEG400 combination was initially used as the solvent for our novel anti-Pseudomonas compounds that we wanted to test for their *in vivo* efficacy in our burn-infection model, the aforementioned dosing schedule was based on the pharmacokinetic properties of our antivirulent compounds.) Every group consisted of 5 mice. Experiments were repeated twice.

### Administration of DMSO-PEG *in vivo* protects the host intestinal barrier function following P. aeruginosa burn site infection.

**FIG 2:**
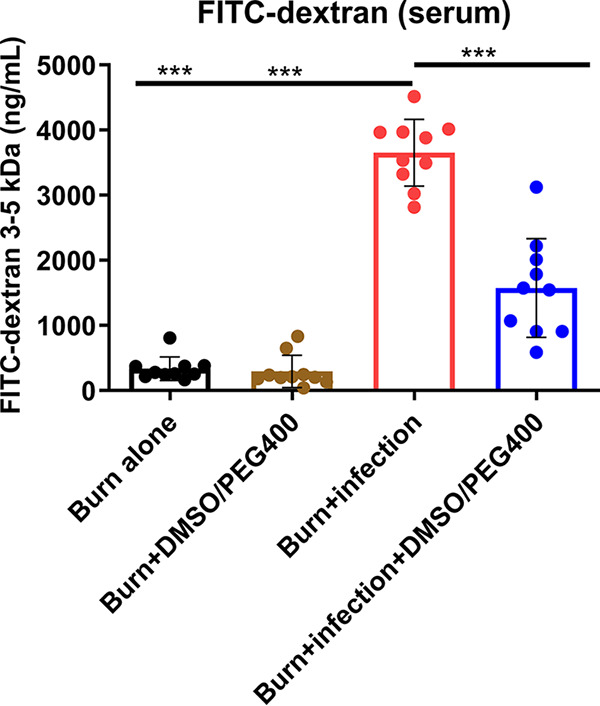
The DMSO-PEG400 combination reduces the FITC-dextran flow from the intestinal lumen to the systemic circulation. The FITC-dextran levels were considerably elevated in the group with burn plus PA14 infection, while DMSO-PEG400 administration significantly reduced these levels. FITC levels were assessed in the serum with fluorescent spectrophotometry (excitation, 480 nm; emission, 520 nm). Data are averages and SEM. Statistical significance was assessed using one-way ANOVA with Tukey's *post hoc* test. ns, not significant; *, *P* < 0.05; **, *P* < 0.01; ***, *P* < 0.001.

Four hours prior to euthanasia, mice were subjected to gavage with 0.2 ml (440 mg/kg) of fluorescein isothiocyanate (FITC)-dextran (3 to 5 kDa; catalog no. FD4; Sigma-Aldrich; Merck KGaA, Darmstadt, Germany) in phosphate-buffered saline (PBS). At 22 h post-BI, mice were euthanized. Blood samples were collected via aseptic cardiac puncture in BD Microtainer SST amber blood collection tubes. Serum was extracted by centrifugation (15,000 × *g*; 90 s) and was used for FITC level assessment (fluorescent spectrophotometry: excitation, 480 nm; emission, 520 nm). The BI mice exerted a higher FITC-dextran flux out of the intestinal lumen (mean FITC-dextran = 3,653.4 ng/ml) than the burn-alone group (mean FITC-dextran = 333.5 ng/ml; *P* < 0.001). DMSO-PEG400 significantly reduced the FITC-dextran flux (mean FITC-dextran = 1,572.2 ng/ml; *P* < 0.001), indicating mitigation of the bacterially mediated derangement of the intestinal permeability (Fig. 2).

### DMSO-PEG400 treatment *in vivo* ameliorates the intestinal inflammation following P. aeruginosa burn site infection.

**FIG 3:**
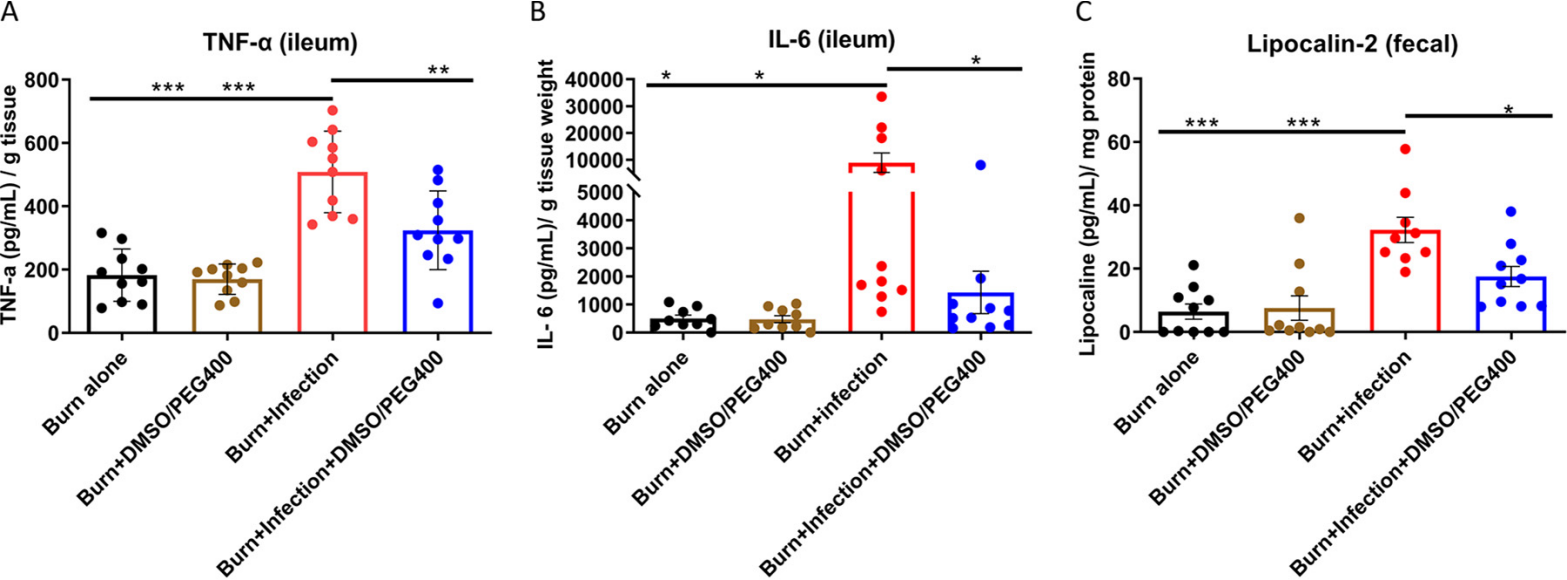
(A) DMSO-PEG400 reduces TNF-α levels in the ileum. The TNF-α levels were considerably elevated in the group with burn plus PA14 infection, while DMSO-PEG400 administration significantly reduced these levels. (B) DMSO-PEG400 reduces IL-6 levels in the ileum. The IL-6 levels were significantly elevated in the burn-PA14 infection group, while DMSO-PEG400 administration statistically significantly reduced these levels. (C) DMSO-PEG400 reduces lipocalin-2 levels in mouse feces. The lipocalin-2 levels were increased in the burn-PA14 infection group, while DMSO-PEG400 treatment significantly reduced these levels. Data are averages and SEM. Statistical significance was assessed using one-way ANOVA with Tukey's *post hoc* test. ns, not significant; *, *P* < 0.05; **, *P* < 0.01; ***, *P* < 0.001.

Deregulation of the intestinal barrier integrity is known to be bidirectionally linked to a heightened intestinal inflammatory response (23, 24). To test the effect of DMSO-PEG400 on the post-BI intestinal inflammation levels, distal ileum samples were aseptically harvested through midline laparotomy, were flushed three times with sterile PBS, and were used for tumor necrosis factor alpha (TNF-α) (mouse TNF-α enzyme-linked immunosorbent assay [ELISA] Ready-SET-Go kit; eBioscience, San Diego, CA, USA) and interleukin 6 (IL-6) (mouse IL-6 DuoSet ELISA; R&D Systems) quantification. The two distalmost stool pellets from the rectum of the mice were also collected, homogenized in sterile PBS, and spun down, and the supernatant was used for the assessment of fecal lipocalin-2 (mouse lipocalin-2/NGAL DuoSet ELISA; R&D Systems). Figure 3A demonstrates a sharp ileal TNF-α rise post-BI (mean TNF-α = 508.2 pg/ml/g) compared to the burn-alone group (*P* < 0.001). DMSO-PEG400 treatment conferred a significant TNF-α reduction (mean TNF-α = 323.9 pg/ml/g; *P* < 0.01). Likewise, DMSO-PEG400 administration also reduced the IL-6 levels in the ileum (mean IL-6 = 8,914 pg/ml/g; *P* < 0.05) (Fig. 3B). Finally, the levels of fecal lipocalin-2, an acute-phase protein that has proven to be a highly reliable marker of the inflammatory status in the intestinal lumen (25), were also reduced following treatment with DMSO-PEG400 (mean lipocalin-2 = 32 pg/ml/mg; *P* < 0.05) (Fig. 3C).

### DMSO-PEG400 treatment *in vivo* attenuates systemic bacterial dissemination.

**FIG 4:**
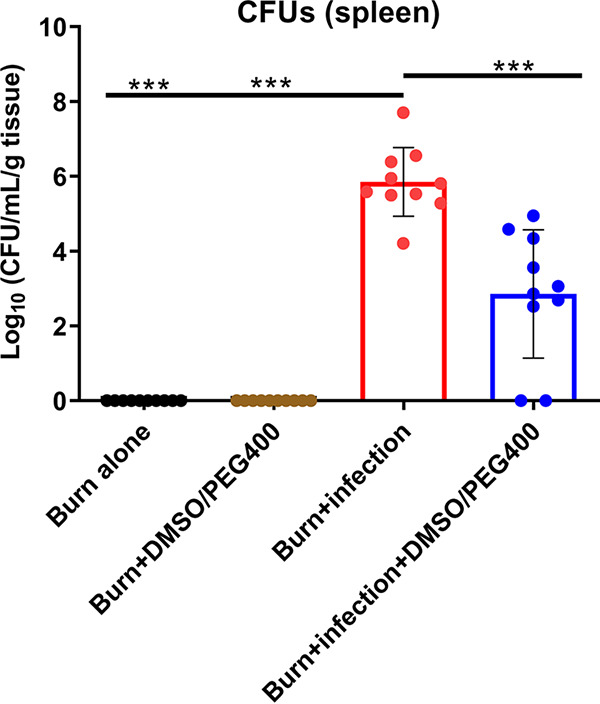
The DMSO-PEG400 combination reduces the systemic bacterial dissemination. The bacterial CFU in the spleen were significantly reduced following DMSO-PEG400 administration compared to those observed in the burn-PA14 infection group. Data are averages and SEM. Statistical significance was assessed using one-way ANOVA with Tukey's *post hoc* test. ns, not significant; *, *P* < 0.05; **, *P* < 0.01; ***, *P* < 0.001.

Subsequently, we tested the effect of DMSO-PEG400 in the post-BI bacterial dissemination from the burn site to the spleen. Spleen samples were aseptically harvested through midline laparotomy and were homogenized in 1 ml sterile PBS using a tissue homogenizer (Polytron, PT 10–35). The homogenates were serially diluted 1/10 to 1/1,000 and plated on LB agar plates. Plates were incubated at 37°C, and CFU were quantified. Figure 4 shows that the spleen bacterial load was significantly higher in the no-treatment group than the DMSO-PEG400 treatment group (*P* < 0.001), indicating an antibacterial role for this solvent combination.

One-way analysis of variance (ANOVA) with Tukey’s *post hoc* comparisons was used for all statistical analyses (GraphPad Prism). *P* values of <0.05 were considered statistically significant.

## DISCUSSION

Previous studies have reported DMSO or PEGs to have antibacterial properties against PA *in vitro* (5–7), and some others have interrogated their anti-PA efficacy *in vivo* (4, 9, 10). Using a posthepatectomy PA infection model, Wu et al. (9) showed that PA grown *in vitro* with PEG3.5 or PEG15-20 prior to *in vivo* infection killed fewer animals than PA grown with 0.9% NaCl. Additional PEG doses administered by gavage further improved animal survival (9). Guo et al. (4) used a burn–PA infection model and found that topical 10% DMSO application on the infected burn site reduced systemic bacterial dissemination as much as the antibiotic control (silver sulfadiazine). Additionally, intraperitoneal 10% DMSO administration improved murine survival (4). These studies show the antibacterial potential of DMSO and PEGs against PA *in vivo*. Our results give further confidence in these solvents' antibacterial efficacy *in vivo* even when their administration takes place away from the site of bacterial inoculation. Simultaneously, we show that they are effective *in vivo* even when bacteria are not grown in a culture that contains the treatment agent itself prior to the animal infection, which would essentially recapitulate an *in vitro* mechanism of action. Li et al. (10) used a zymosan-induced acute peritonitis model in rats and showed that 5% DMSO administration improves the intestinal inflammatory state and barrier function (10). Our study further strengthens this notion by showing that DMSO-PEG400 can improve intestinal permeability and inflammation even in the setting of infection-induced rather than chemically induced dysfunction.

DMSO and PEGs are currently part of drug formulations approved by the Food and Drug Administration (FDA). A 50% (wt/wt) DMSO solution is available for the symptomatic relief of interstitial cystitis (https://www.ichelp.org/diagnosis-treatment/treatments/bladder-instillations/dmso/), while DMSO is the solubilizing excipient in several drug products (26, 27) (https://www.accessdata.fda.gov/scripts/cder/iig/index.cfm?event=BasicSearch.page, search for inactive ingredient name: dimethyl sulfoxide). Likewise, PEG3350 is an FDA-approved laxative (https://www.drugs.com/pro/polyethylene-glycol.html). PEGylation is used in several therapeutics to improve their pharmacokinetic and pharmacodynamic properties (28) (https://www.biochempeg.com/article/58.html). PEG400, in particular, is an inactive ingredient in 64 FDA-approved drug formulations (https://www.accessdata.fda.gov/scripts/cder/iig/index.cfm?event=BasicSearch.page, search for inactive ingredient name: polyethylene glycol 400).

The toxicology of these solvents has been studied extensively, with no particular concerns (29, 30). Nonetheless, these solvents may exhibit other effects that can potentially influence the outcome of an assay or a system being tested. Given their antimicrobial activity, it is imperative to be extremely cautious when they are used in compound formulations that one intends to test for their antimicrobial efficacy. One of the most attractive, novel, alternative antimicrobial approaches is that of targeting bacterial virulence. In this effort, selecting appropriate solvents for the newly tested compounds is of utmost importance. Antivirulent compounds are not expected to have bactericidal or bacteriostatic activity. Therefore, it is crucial to solubilize these compounds in solvents with no antibacterial activity to avoid concealing the drug’s antivirulence efficacy. Such solvents include, but are not limited, to Captisol aqueous solutions, hydroxypropylmethylcellulose (HPMC), carboxymethylcellulose, 2-hydroxypropyl-β-cyclodextrin (2HPβCD), α-cyclodextrin (α-CD), corn oil, sesame oil, and cottonseed oil. Other nonaqueous solvents may also prove to possess antibacterial effects on different microorganisms. Incorporating the appropriate controls is of utmost importance to ensure that the solvent does not affect the experiment's final results.
